# An empirical approach to assessing training needs for emergency department management of intentional self-harm and related behaviors in the United States

**DOI:** 10.3352/jeehp.2017.14.30

**Published:** 2017-12-14

**Authors:** Monica Whitehead, Jeffrey Shahidullah, Paul Kettlewell, Nicole Quinlan, Robert Strony

**Affiliations:** 1 Geisinger Health System, Danville, PA, USA; 2 Rutgers University and Robert Wood Johnson Medical School, New Brunswick, NJ, USA; Hallym University, Korea

The emergency department (ED) is the only healthcare setting that guarantees a same-day mental health evaluation, making this location a viable option to patients in imminent need. As such, the ED is frequently the first place of contact for patients to seek and/or receive care for mental health needs [1]. Despite the prevalence of mental health concerns that emergency medicine residents are expected to manage, residents often receive inadequate training in this area [2,3]. There is a need for empirical approaches to evaluate training needs and progress. Moving forward, it would be helpful for studies to assess detailed aspects of care for which residents may report that they need more targeted training. This data-based approach to improvement would ensure that the often-limited resources of residency training programs (e.g., time and faculty allotment) can best be used to improve the capacities in the greatest need of support. The primary purpose of this paper is to demonstrate how training programs can use a short, empirically-driven survey to evaluate their training needs. We hypothesized that such an instrument would yield a breadth of information applicable for assessing our training program’s needs. This study presents the approach of an emergency medicine residency training program to develop an instrument for the evaluation and formative assessment of emergency medicine residents throughout the course of residency. We present data from this training program’s first iteration of using this instrument to assess current attitudes towards and knowledge of patients with mental health-related issues.

## Participants

This study was cross-sectional in nature. The participants were first, second, and third post-graduate year (PGY 1–3) emergency medicine residents of Geisinger Medical Center, in the United States (PGY 1, n=17; PGY 2/3, n=13). Due to the relatively small sample size, PGY 2 and PGY 3 residents were combined. The data were collected in July 2016 and July 2017, the beginning of each training year. Table 1 presents the demographic and training variables.

## Procedures

This study was reviewed by the local Institutional Review Board of Geisinger Medical Center and determined to be exempt (IRB # 2016-0283) after receiving informed consent from the subjects. Surveys were administered to emergency medicine residents at the beginning of the training year. The instructions were read by a research assistant, and included information about the voluntary nature of this study, how data would be de-identified, and how data would not be used for formal performance evaluations. Once completed, names were replaced with a study identification number. Only the research assistant had access to the link between the name and identification number.

## Measure

Two domains were assessed: residents’ self-reported confidence and their competence/accuracy when answering knowledge-based questions. A 19-item survey was developed by study investigators due to the lack of validated surveys in the literature (Appendix 1). Items 1–10 consisted of questions pertaining to demographics and training history, and items 11–14 consisted of residents’ self-reported confidence in 4 domains rated on a Likert-type scale of 1–10 (1= not at all confident to 10= very confident). Item 15 was an open-ended question asking residents what they would like to learn more about to effectively address psychiatric issues. Items 16–19 consisted of open-ended questions regarding competence in mental health-related issues. The competence items were scored based on the American Academy of Child and Adolescent Psychiatry’s practice parameters for the assessment and treatment of children and adolescents with suicidal behavior [4] and local and hospital laws. Doctorate-level clinical psychologists assisted in the generation and scoring of this instrument. Since a scoring rubric was used, no biases were anticipated in scoring. Raw data are available in Supplement 1.

The 2 groups (PGY 1 versus PGY 2/3) were evaluated separately to allow for comparisons and to determine the degree to which learning improved through residency. The independent-samples t-test was used to examine differences

The independent-samples t-test was used to examine residents’ confidence in their mental health knowledge and skills. Confidence was assessed for the following 4 domains: conducting a mental status exam, identifying provisional mental health disorders, conducting risk assessment evaluations, and developing a follow-up plan. Residents’ confidence ratings ranged between 6.43 and 7.13 on a scale of 0 to 10 (Table 2). Differences were found in respondents’ confidence related to developing a follow-up plan (t [28] = 2.77, P= 0.01), with PGY 2/3 residents endorsing more confidence than PGY 1s.

The independent-samples t-test was used to examine competence related to mental health crises (Table 3). For identifying risk factors related to intentional self-harm, out of a possible score of 12, residents identified 3–8 risk factors, with an average of 4.90 factors. In terms of developing a safety plan, out of a possible 4 points, scores ranged from 0 to 3, with an average score of 1.90. PGY 2/3 residents could identify more pertinent elements of a safety plan than PGY 1 residents (t [28] = 2.26, P= 0.03). When differentiating between voluntary and involuntary admissions paperwork, out of a possible 3 points, residents scored 0–2 points, with an average of 0.60 points. PGY 2/3 residents more accurately identified who signs hospital admission paperwork, the length of hold, and the consequences of these admissions than PGY 1 residents (t [28] = 7.51, P< 0.001). In practice vignettes related to signing paperwork, out of a possible score of 5 points, scores ranged from 0 to 5, with an average of 2.13 points. On the item requiring respondents to describe the importance of toxicology screenings, scores ranged from 0 to 2 out of a possible 2 points, and the average score was 0.93 points.

From an internal quality improvement perspective, emergency medicine departments can use the instrument described herein in a number of useful ways to inform their training efforts. Training faculty can ascertain the specific skills in which trainees demonstrate marked improvement and which skills remain stagnant. We suggest that these data may be useful for generating targeted training initiatives to remediate areas of low performance that do not sufficiently improve. Of course, decisions regarding what constitutes a sufficient score in a given competency will be made on an individual program basis.

As an example, the data described in this paper show that PGY 2/3 residents displayed significantly higher levels of confidence than PGY 1 residents in developing a follow-up plan in response to patients endorsing suicidality. However, confidence in other areas related to psychiatric crises did not significantly differ between the groups. This data, along with other sources of information such as feedback solicited from residents and faculty, may indicate that innovations in the training curriculum are necessary, such as more frequent experiential encounters with experienced faculty. As another example, our data demonstrated that there were no between-group differences in competencies relating to signing psychiatric admissions paperwork as assessed through vignettes, but differences were found when participants were asked to list a set of facts. As such, we believe that our data support the use of observations and live performance feedback for our residents. Therefore, our hypothesis that we could obtain a breadth of useful information to make training decisions was confirmed. In addition to identifying areas for improvements in training, programs can quantitatively evaluate the effectiveness of various training interventions by comparing results after a training intervention with a baseline pre-training score or with the scores of a group that did not receive the intervention.

The utility of this training evaluation approach should be viewed in the context of a key limitation. Without psychometric validation of the assessment measure, firm conclusions regarding the efficacy of the training curriculum cannot be made. Our data may reflect differences between cohorts rather than true improvement over time. The existence of similar scores between groups does not necessarily mean that no learning/improvement in mental health took place. Rather, the domains that we specifically assessed may not have improved. Nonetheless, this assessment approach can be used with ongoing iterative feedback to track responses to changes in the curriculum. Although not generalizable in the sense of allowing assumptions to be made about how residents would perform at other sites, the data from this assessment may be useful for programmatic decision-making purposes to ensure that training opportunities are standardized and structured in response to identified needs.

## Figures and Tables

**Table 1.** Demographic and training information of participants

Characteristic	All	PGY 1	PGY 2/3
No. of emergency medicine residents	30	17	13
Average age of residents (yr)	29.97 ± 3.55	29.19 ± 2.93	30.92 ± 4.11
Female	8 (26.7)	7 (41.2)	1 (7.7)
Foreign medical education	1 (3.3)	1 (5.9)	0
Osteopathic medical school (compared to allopathic)	19 (63.3)	10 (58.8)	9 (69.2)
Natural or life sciences major	27 (90.0)	15 (88.2)	12 (92.3)
Humanities major	3 (10.0)	2 (11.8)	1 (7.7)
Medical school training in psychiatry or other behavioral health setting (wk)	4.97 ± 1.40	5.24 ± 1.48	4.62 ± 1.26

Values are presented as mean±standard deviation or number (%). PGY, postgraduate year. ^*^P<0.05, ^**^P<0.01 for differences between PGY 1 and PGY 2/3 residents.

**Table 2.** Comparison of confidence ratings of practice parameters across years

Evidence-based practice parameter	Score range	Average score		
		All	PGY 1	PGY 2/3
Mental status exam	3–10	6.70 ± 1.60	6.47 ± 1.81	7.00 ± 1.29
Diagnosing mental disorders	3–9	6.23 ± 1.46	6.29 ± 1.40	6.15 ± 1.57
Risk assessment evaluation	4–10	7.13 ± 1.48	6.82 ± 1.51	7.54 ± 1.39
Plan for follow-up	3–9	6.43 ± 1.38	5.88 ± 1.27	7.15 ± 1.21^**^

Values are presented as mean±standard deviation. Confidence scores ranged from 1 (least confident in knowledge of evidence-based practices) to 10 (most confident in knowledge of evidence-based practices). PGY, postgraduate year. ^*^P<0.05, ^**^P<0.01 for differences between PGY 1 and PGY 2/3 residents.

**Table 3.** Comparison of knowledge of practice parameters across years

Evidence-based practice parameter	Score range	Average score		
		All	PGY 1	PGY 2/3
Risk factors for intentional self-harm (0–12)^a)^	3–8	4.90 ± 1.27	4.82 ± 1.19	5.00 ± 1.41
Intentional self-harm safety plan (0–4)^a)^	0–3	1.90 ± 0.92	1.59 ± 0.87	2.31 ± 0.86^*^
Increased supervision				
Removing means				
Follow-up				
Caution family about disinhibiting effects of alcohol/drugs				
Differences between voluntary and involuntary psychiatric admission (0–3)	0–2	0.60±0.77	0.06±0.24	1.31±0.63^**^
Who signs/for what ages				
Length of hold				
Long-term consequences				
Vignettes on voluntary versus involuntary psychiatric admission (0–5)	0–5	2.13±1.80	1.65±1.54	2.77±1.96
Five vignettes				
Importance of toxicology screens and psychiatric admissions (0–2)	0–2	0.93±0.58	0.88±0.70	1.00±0.41
Accurate assessment				
Informed consent				

Values are presented as mean±standard deviation. PGY, postgraduate year.

a) Based on the American Academy of Child and Adolescent Psychiatry practice parameters for suicidal behavior.

* P<0.05,

** P<0.01 for differences between PGY 1 and PGY 2/3 residents.

## Supplementary materials

Supplement 1. Data file is available from https://doi.org/10.7910/DVN/TVCZFU.
