# Supplementary material for: An empirical approach to assessing training needs for emergency department management of intentional self-harm and related behaviors in the United States
Source: J Educ Eval Health Prof. 2017 Dec 14;14:30. doi: 10.3352/jeehp.2017.14.30 (PMC5801318; doi:10.3352/jeehp.2017.14.30)
Supplement: Supplementary file 1 [file jeehp-14-30-app1.pdf]

Appendix 1. The emergency room behavioral health survey administered to emergency medicine residents

1. Full name:
2. What year were you born?
3. Current residency year?
4. What is your sex?
5. What was your major in your undergraduate training?
6. Did you complete your medical school in the United States?
7. What year did you complete medical school?
8. Did you complete an allopathic or osteopathic medical school?
9. How many weeks did you spend working in psychiatry or other behavioral health settings during your medical school training?
10. Outside of medical school, have you ever worked (volunteer or paid) in a mental health or substance abuse setting before?

Please indicate your confidence level (scale of 1–10; 1 = not at all confident, 10 = very confident) on your ability to:

11. Conduct a mental status examination (assessing for appearance and general behavior, motor activity, speech, mood and affect, thought processes, thought content, perceptual disturbances, sensorium and cognition, insight, and judgement)?
12. Establish a provisional diagnosis(es) of the mental disorder(s) most likely to be responsible for the current emergency, including identification of any general medical condition(s) or substance use that is causing or contributing to the patient's mental condition?
13. Conduct a risk-assessment evaluation (may include consideration of suicide or homicide risk as well as other forms of self-injury [e.g., cutting behaviors, accidents], aggressive behaviors, neglect of self-care, or neglect of the care of dependents) with consideration for risk and protective factors?
14. Develop a specific plan for follow-up, including immediate treatment and disposition; determine whether the patient requires treatment in a hospital or other supervised setting and what follow-up will be required if the patient is not placed in a supervised setting?
15. In the next year, what would you like to learn more about in order to effectively address psychiatric issues in the emergency room? (open-ended)

Please provide a free response to the following clinical scenarios or questions:

16. A young patient is identified with signs or symptoms suggesting suicidality. Please list key risk factors and list the steps in developing a suicide safety plan.
17. Describe the difference between a voluntary psychiatric admission and an involuntary psychiatric admission. In your answer describe who can sign each of these admission documents for admission for patients 18 and older, and for those under 18.
18. For each of the following scenarios regarding possible psychiatric admission after presentation to the ED, indicate whether this should be voluntary or involuntary, and who would sign it:
    a) A 16-year-old attempted to swallow a bottle of pills, but his mother stopped him. She would like him admitted, but he "doesn't want help" and does not want admission.
    b) A 25-year-old is having suicidal thoughts and has formulated a plan, but would like to be admitted to keep her from following through with the plan.
    c) A 67-year-old told his family that he was going to kill himself and no one was going to stop him. He has several firearms at home in addition to a car and a garage. His daughter brought him to the ED and would like him admitted. He confirms daughter's statements, but otherwise refuses to engage in any evaluation.
    d) A 14-year-old reports feeling depressed, has self-harmed, and thought about walking into traffic. She keeps thinking about doing this. She would like to be admitted, but her father would prefer to just take her home with some new medicine.
    e) A 13-year-old beat up his sister, held a knife to his mother's throat and tied a rope around his own neck. He is cursing and screaming in the ED and refusing to be admitted. Mother does not feel safe taking him home.
19. Why is it important to have toxicology screens (including alcohol) completed—and, if applicable, patient "sober up"—prior to admission decisions, including signing of paperwork?

EM, emergency medicine.
